# Efficiency of platelet-rich plasma in management of oral lichen planus: a systematic review and meta-analysis

**DOI:** 10.21142/2523-2754-1403-2026-304

**Published:** 2026-07-10

**Authors:** Radhika Sasikumar, Amita Aditya, Vaishnavi Sawant, Neha Kulkarni, Alisha Fargade, Vaishnavi Diwan

**Affiliations:** 1 Department of Oral Medicine & Radiology, Dr. D.Y. Patil Dental College & Hospital, Dr. D. Y. Patil Vidyapeeth, Pimpri, Pune.radika.ammusasi@gmail.com dr.amitaaditya@gmail.com vssavant1@gmail.comknehan999@gmail.comishafargade@gmail.comvaishnavi.diwan2001@gmail.com Department of Oral Medicine & Radiology Dr. D.Y. Patil Dental College & Hospital Dr. D. Y. Patil Vidyapeeth Pimpri Pune radika.ammusasi@gmail.com dr.amitaaditya@gmail.com vssavant1@gmail.com knehan999@gmail.com ishafargade@gmail.com vaishnavi.diwan2001@gmail.com

**Keywords:** lichen planus, platelet rich plasma, therapy, growth factors, liquen plano, plasma rico en plaquetas, terapia, factores de crecimiento

## Abstract

**Background:**

Over the years, studies investigating platelet-rich plasma as a future therapy for oral lichen planus have been on the rise. However, thorough evaluation is required to determine its efficacy, since various studies are being carried out on diverse population with long-term follow-ups.

**Objective:**

To evaluate the current evidences to analyze the efficiency of platelet-rich plasma as a treatment modality for oral lichen planus.

**Material and Methods:**

This review adhered to PRISMA 2020 guidelines. Studies published within the last eight years were screened across multiple databases such as PubMed, Scopus, Google Scholar, and Science Direct. The studies eligible were: randomized controlled trials; cohort; and longitudinal designs comparing PRP with other treatment modalities of which six clinical studies were included which had 129 participants in total. Cochrane Risk of bias (RoB) tool was used for Visual analog scale values were pooled for meta-analysis. Meta-analyses were performed using fixed-effect models.

**Results:**

Clinically, both control and intervention groups exhibited a pooled mean difference of 0.61, 95% CI (0.22-0.99). As there were variations within the studies, the fixed effects model was chosen for analysis. Comparative analysis of the results demonstrated that both the platelet-based therapies and the topical corticosteroid approach improved patient satisfaction and comparably reduced pain and lesion size in individuals with erosive OLP in similar measures**.**

**Conclusion:**

Oral lichen planus can be safely and perhaps effectively treated with platelet-rich plasma. Future studies should adopt a standardised approach in evaluating efficacy of PRP in OLP. Rigorous clinical trials less heterogeneity in the methodology is required for high-quality evidence.

## RESUMEN

**Antecedentes:**

Con el paso de los años, han aumentado los estudios que investigan el plasma rico en plaquetas como posible terapia para el liquen plano oral. Sin embargo, se requiere una evaluación exhaustiva para determinar su eficacia, ya que se están realizando diversos estudios en distintas poblaciones con seguimiento a largo plazo.

**Objetivo:**

Evaluar la evidencia actual para analizar la eficacia del plasma rico en plaquetas como modalidad de tratamiento del liquen plano oral.

**Materiales y métodos:**

Esta revisión se ajustó a las directrices PRISMA 2020. Se revisaron estudios publicados en los últimos ocho años en múltiples bases de datos como PubMed, Scopus, Google Scholar y ScienceDirect. Los estudios elegibles fueron: ensayos controlados aleatorizados; estudios de cohorte; y diseños longitudinales que comparaban el PRP con otras modalidades de tratamiento. Se incluyeron seis estudios clínicos con un total de 129 participantes. Se utilizó la herramienta Cochrane de riesgo de sesgo (RoB) para agrupar los valores de la escala analógica visual para el metanálisis. Los metanálisis se realizaron utilizando modelos de efectos fijos.

**Resultados:**

Clínicamente, tanto el grupo control como el grupo de intervención mostraron una diferencia media agrupada de 0,61 (IC del 95%: 0,22-0,99). Debido a las variaciones dentro de los estudios, se optó por el modelo de efectos fijos para el análisis. El análisis comparativo de los resultados demostró que tanto las terapias basadas en plaquetas como el enfoque con corticosteroides tópicos mejoraron la satisfacción del paciente y redujeron de forma comparable el dolor y el tamaño de la lesión en personas con LPO erosivo en medidas similares.

**Conclusión:**

El liquen plano oral puede tratarse de forma segura y, posiblemente, eficaz con plasma rico en plaquetas. Los estudios futuros deberían adoptar un enfoque estandarizado para evaluar la eficacia del PRP en el LPO. Se requieren ensayos clínicos rigurosos, con menor heterogeneidad metodológica, para obtener evidencia de alta calidad.

## INTRODUCTION

Oral lichen planus (OLP) is an autoimmune condition that impacts the oral mucosa, with a global prevalence estimated between 0.5% and 2.2%. ^1^ Furthermore, patients' quality of life is substantially impacted by the atrophic and erosive variants, which typically exhibit the most symptoms, including excruciating pain and a burning sensation. Beyond that, OLP poses a 0.5% to 2% risk of malignant transformation, which is particularly prevalent in middle-aged women (female-to-male ratio of 2:1). ^2^ The essential search for a definitive intervention that achieves a complete and sustained cure for OLP remains a primary concern. ^3^

The mainstay therapy of OLP has been the topical corticosteroids which is due to their anti-inflammatory effectiveness. ^4^ However, prolonged use of corticosteroids has been attributed substantial side effects such as dysgeusia, tachyphylaxis, mucosal atrophy, and secondary candidiasis thereby a growing interest in other remedies other than conventional corticosteroid therapy, such as remedies like dapsone, antimalarials, interferon, hyaluronic acid, PUVA therapy, and platelet-rich plasma (PRP). ^5^^,^^6^

In the past decades, PRP has evolved into a promising supplemental therapy. PRP is an autologous concentrate which is comprised of potent angiogenic and mitogenic factors which synergistically facilitate cell proliferation, chemotaxis, angiogenesis, and tissue regeneration.^7^ It assures more effective biocompatibility, reducing the likelihood of allergic or immunological reactions, while its anti-inflammatory characteristic facilitate the reduction of related morbidity and expedite tissue repair. ^8^

Although there have been investigations conducted for the use of PRP in managing the oral lesions, but there are limited evidence-based studies including Randomized controlled trials. There also seems to be clinical uncertainty due to the paucity of data regarding the long term side effects and prolonged follow-up periods. ^9^

This systematic review focusses on synthesis of the existing evidence, serving as the foundational pre-requisite to further develop evidence-based guidelines to explore the efficiency of PRP in managing OLP.

## MATERIALS AND METHODS

### Protocol and Registration

This review strictly adhered to the guidelines provided by the Cochrane Handbook and PRISMA 2020 checklist and has been registered in the international prospective register of systematic reviews, PROSPERO (CRD420251158089).

• Population: Patients with OLP
• Intervention: Platelet-rich plasma in the form of intralesional injections
• Comparator: Intra-lesional corticosteroid injections
• Outcomes: Changes in symptoms and reduction in area of the lesion.

### Search Strategy

A broad search was executed in the Google Scholar, PubMed, Scopus, and Science Direct sources to discover articles of interest. 

### Study Selection and Eligibility

**Figure 1:**
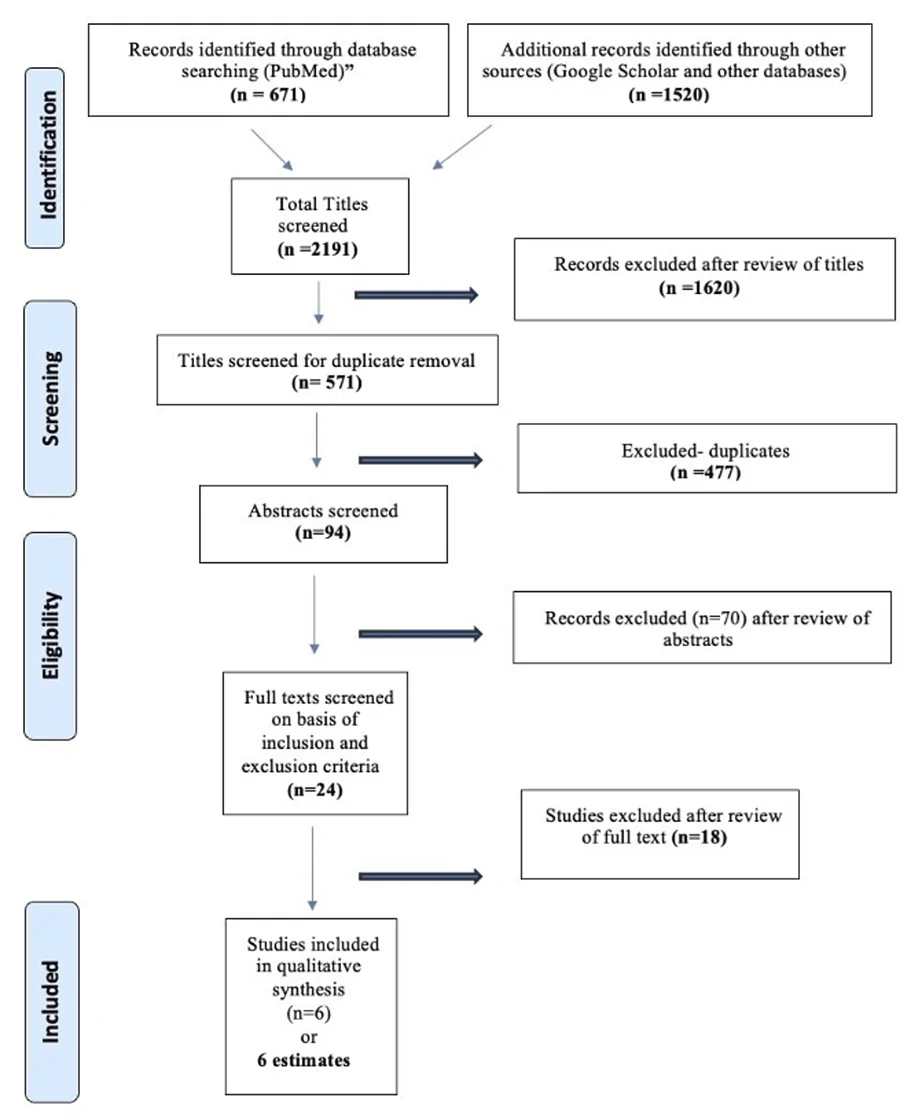
Showing identification of studies via database and register

The literature search adhered to a rigorous, three-step screening process as shown in figure 1. Trials and studies were assessed by one reviewer initially and second reviewer arbitrated any ambiguity in the selection. Clinical studies and split mouth studies containing adequate details regarding the use of PRP as a cure for oral lichen planus, included a mean and standard deviation in their findings, published between 2016 and 2024 and available in full text in English were considered eligible. Letter to editor and editorial, short communication, reviews, animal studies and invitro studies were excluded.

### Data Extraction and Risk of Bias

Study characteristics, author name, year of publication, study design, clinical parameters, intervention, follow-up period, and sample size were among the data extracted. Duplicates were eliminated using Rayyan QCRI software, and the extracted data was saved in Microsoft Excel 2013. The Cochrane risk of bias (RoB) 2 tool was used for RoB assessment and “Traffic Light” plot was created using ROBVIS tool for its visual representation. 

### Quantitative Data Synthesis 

The study's outcome, pain determined by VAS scores, was quantitatively summarized by meta-analysis. Four of the six studies that included for the systematic review reported this specific outcome. Thus, a meta-analysis of VAS scores was conducted. The findings for each study's intervention group and control group are shown in Table 1 as the mean and SD.

**Table 1:** Quantitative data depicting Mean and SD values of Pain: VAS scores in intervention group (PRP/PRF) and control group (Steroid/TA) amongst included studies

No.	Included studies	Intervention group (PRF/PRP)			Control group (Steroid/TA)		
		Mean	SD	Total	Mean	SD	Total
1	Saglam et al.	19.79	18.15	24	20.83	17.61	24
2	Bennardo et al.	2.9	2.1	9	1.9	1.5	9
3	Sharma et al.	0.7	0.865	20	0.1	0.3	20
4	Elghareeb et al.	3	2.66	12	2.58	3.45	12

## RESULTS

The primary objective of the current systematic review was to evaluate the clinical success rate of platelet-rich plasma (PRP) and platelet-rich fibrin (PRF) in the treatment of Oral Lichen Planus (OLP). A total of six studies met the predetermined inclusion criteria for the systematic review (summarized in Table 2).

**Table 2:** Details of the studies included in the systematic review

Study Id	Author	Year	Study design	Sample size
1	Saglam et al.	2021	a split-mouth, randomized, controlled clinical trial	n = 24
2	Bennardo et al.	2021	a prospective randomized split-mouth study	n = 9
3	Hijazi et al.	2022	A pilot randomized clinical trial	n = 20
4	Al-Hallak et al.	2022	split-mouth randomized trial	n = 12
5	Sharma et al.	2023	randomized controlled clinical study	n = 40
6	ElGhareeb et al.	2023	A pilot randomized clinical trial	n = 24

Key study characteristics, including age groups, specific interventions, comparators, and primary outcomes, are detailed in Table 3. Patients included in the research typically presented with a clinical picture indicative of EOLP, characterized by bilateral, roughly symmetrical erosive lesions often surrounded by a lacelike network of grey-white striations. In most protocols, the test group received 1-ml intralesional injections of PRF or PRP into one side of the buccal mucosa. The comparator (control) group, conversely, received an injection of 40 mg of triamcinolone acetonide (TA) combined with 2% lignocaine hydrochloride on the contralateral side of the lesion. Follow-up periods averaged three months, generally ranging from a minimum of fifteen days to a maximum of six months.

**Table 3:** Details of the study participants, intervention, and comparator of the studies included in the systematic review

No.	Author	Age group	Population	Interventions/treatments used (Test group)	Comparator (Control group)	Follow-up period	Primary outcomes	Results	Conclusion
1.	Saglam et al.	34 and 76 years old	individuals diagnosed histopathologically with bilateral EOLP.	One bilateral lesion was injected with i-PRF,	other was injected with methylprednisolone acetate	four sessions at 15-day intervals, 6 months follow-up	Visual analog scale (VAS) for pain and satisfaction, oral health impact profile scale-14, and the lesion size were used	The intragroup comparisons showed a significant decrease in VAS-pain and lesion size in both the i-PRF group. However, no significant difference in any value occurred in the intergroup comparisons.	In patients with Erosive Oral Lichen Planus, both methods decreased pain and lesion size similarly, and both increased satisfaction. Therefore, the use of i-PRF may be considered an option in cases refractory to topical corticosteroid therapy. Biochemical and histopathological studies are required to reveal the mechanism of i-PRF action in EOLP treatment.
2.	Bennardo et al.	55-64years	Participants with symptomatic OLP were recruited	patients randomly received a 0.5-mL TA injection in one buccal mucosa	1-mL PRF injection in the opposite side.	1 month	reduction of the lesions area and symptom atology modifications using visual analogue scale (VAS) score	Four weeks after the last injections, an average reduction of 59.8% in the lesion extension and an average reduction of 47.6% in the VAS score for PRF-treated sites were observed; the same variation for TA-treated sites was respectively of 59.2% and 40%. There were no statistically significant differences between the two groups.	Platelet-rich fibrin (PRF) was effective in reducing OLP lesions extension and symptomatology, and it seems to be as effective as triamcinolone acetonide (TA)
3.	Hijazi et al.	18-70 years	clinical picture that suggests the diagnosis of erosive OLP; bilateral, more or less symmetrical erosive lesions with a lacelike network of slightly raised graywhite lesions	Patients received weekly intralesional injections of PRP for 4 weeks	Patients received weekly intralesional injections of TA for 4 weeks	3 months	Pain scores using numerical pain score and clinical score	Both groups showed significant improvement in the clinical parameters (pain and clinical score) “p=.001. However, there is no statistical significance when comparing the two groups in pain score, clinical score, or remission.	PRP injections could be considered as an effective alternative single treatment modality for EOLP
4.	Al-Hallak et al.	35-60 years	patients with symptomatic, bilateral OLP lesions.	1-ml intralesional PRF injection on one side of the buccal mucosa	0.5-ml TA injection on the counterpart side	once a week for 4 weeks.	visual analog scale score, REU score, and lesion areas.	Both injectable TA and PRF were effective in the management of oral lichen planus. After 4 weeks of treatment, there was an average reduction in the VAS score and an average reduction in the REU score. There were no statistically significant differences between the two treatment methods	Intralesional injection with TA showed more effectiveness than i-PRF in the management of OPL lesions. Although, i-PRF cannot be considered a first-line treatment option, it showed promising alternative therapy choice with no side effects.
5.	Sharma et al.	18-60 years	Patients visiting dental OPD diagnosed clinically with either erosive lichen planus or OSMF.	Autologous PRP was prepared and injected on the other side in same patients	Triamcinolone 40 mg (1mg/ml) mixed with 2% (1:80,000) lignocaine hydrochloride was injected into the lesion on one side	4 months	all the patients were evaluated for pain and burning sensations, size, and severity of the lesion, and interincisal mouth opening (mm).	Intralesional injections of triamcinolone acetonide and PRP are effective in reducing pain and burning sensation in OSMF, but PRP is less effective in improving cheek flexibility as compared to triamcinolone acetonide. In OLP, both triamcinolone acetonide and PRP are almost equally effective in reducing the size of the lesion.	PRP is an effective modality in treating both OSMF and oral erosive lichen planus with no adverse effects.
6.	ElGhareeb et al.	40 to 60 years	participants with clinically diagnosed as OLP were enrolled	PRP Injection was done at four point of the lesional periphery:	intralesional injection of triamcinolone acetonide 40 mg/ml vail mixed with 1 ml of lidocaine 2% with 20 mg/ml of TA final concentration	2 weeks for 4 months	reduction of lesional areas, REU scores, and NRS scores	There was a statistically significant decrease in REU and pain score in both groups after treatment compared to before.	Intralesional PRP is a good and safe modality for treatment of OLP and in tralesional TA. However, there were some side effects and recurrence of disease after follow- up for three months in patients treated by PRP more than those treated by TA.

**Figure 2:**
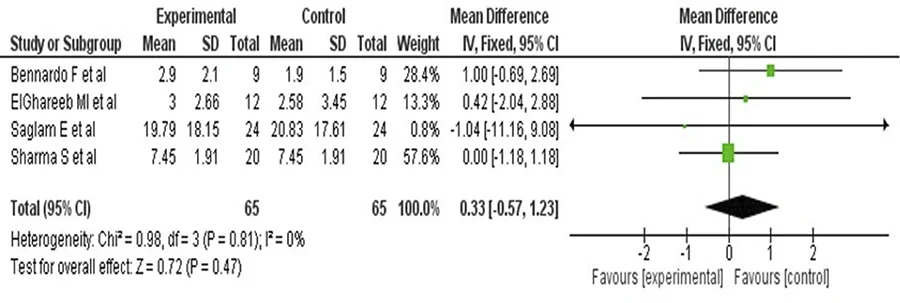
Forest Plot Distribution for Outcome: Pain - Mean VAS scores

The forest plot in Figure 2 displays the intervention/experimental group on the left side versus the comparator/control on the right. The two groups' mean VAS scores were nearly similar. Hence, it can be inferred that both the platelet-based therapies and the topical corticosteroid improved patient satisfaction and comparably reduced pain and lesion size in individuals with erosive OLP in similar measures.

### Risk of Bias

The six included studies had an overall low risk of bias, according to the RoB2 tool, with the exception of D3, which was the subject of some concerns due to missing outcome data from two studies. (Figure 3a and Figure 3b).

**Figure 3a:**
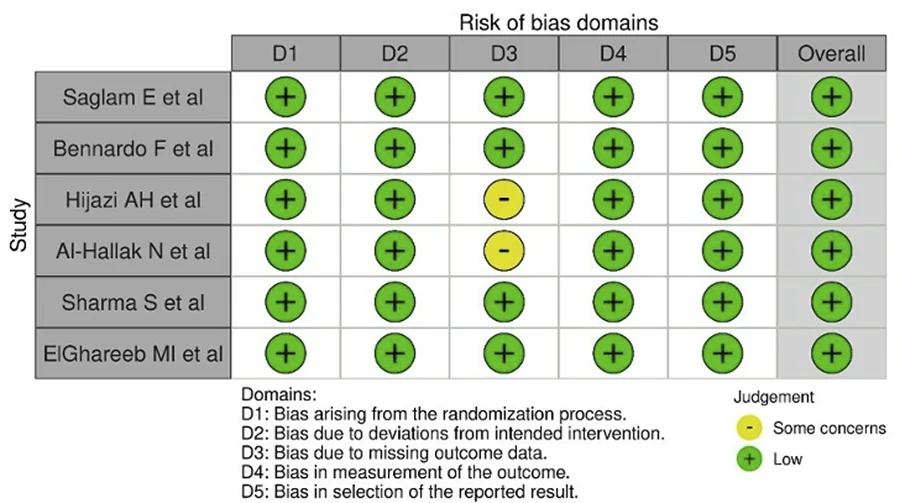
Risk of bias traffic light plot using RoB-2 tool

**Figure 3b:**
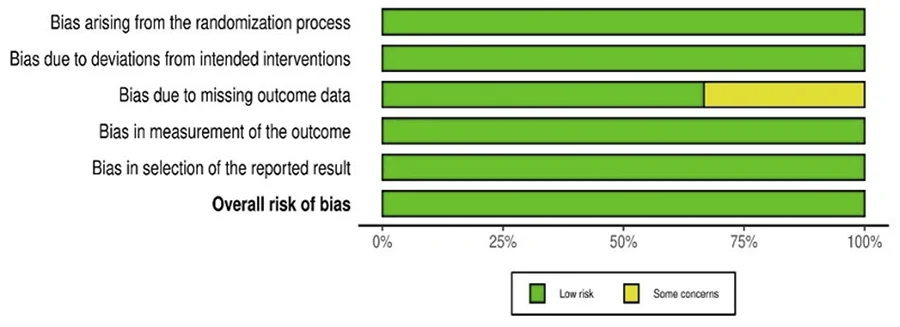
RoB2 “Summary Plot” distribution of risk of bias among the studies

## DISCUSSION

The findings of our systematic review and metanalysis indicate that PRP holds promising value as a therapy to alleviate pain as well as enhance mucosal healing among those who have OLP, particularly those cases where first-line therapies have been unsuccessful or caused harmful side effects.

Our meta-analysis implies that PRP would provide important symptomatic benefit, ideally in the erosive or atrophic forms of oral lichen planus (OLP). These clinical outcomes underline the already established biological activities of PRP. ^9^^,^^10^

In addition to pain relief, some of the included studies also yielded positive outcomes on lesion size reduction and mucosal healing. Again, since measurement methods as well as scores varied, these results could not be combined quantitatively. Recurrence rates post-PRP therapy also were not consistently reported among the studies, so we could conclude little about the long-term persistence of the effect achieved. ^11^ There was one study that reported a statistically significant superiority for the injectable PRF group over the steroid group. Conversely, a separate included study noted that patients treated with PRP exhibited higher rates of disease recurrence and adverse effects compared to those treated with intralesional triamcinolone acetonide (TA) after three months of follow-up. PRP also potentially acts to help mucosal regeneration rather than inflammation suppression, suggesting a new, yet potentially adjunct, mechanism of action. ^12^^,^^13^

Most studies included within the analysis, as well as previous independent clinical studies and case reports, suggest that Platelet-Rich Plasma (PRP) yields significant improvements in pain relief, lesion size decrease or mucosal healing, as well as global patient satisfaction and quality of life. ^14^^-^^17^

Results of our review are consistent with earlier reports that emphasize PRP's autologous nature and low immunogenicity. ^18^^-^^20^ However, it was also noted that the included studies had small sample sizes, with lack of blinding or placebo controls along with shorter follow-up period. There was wide variation in PRP preparation methods (single spin vs. double spin), platelet concentration and its application technique (topical vs. injectable). ^21^^-^^24^ This makes direct comparisons and pooled analysis more challenging. 

### Clinical Implication

Our systematic review and meta-analysis suggest that Platelet-Rich Plasma (PRP) yields significant improvements in pain relief (measured by Visual Analog Scale (VAS) scores), lesion size decrease or mucosal healing, as well as global patient satisfaction and quality of life. ^25^^,^^26^ This is similar to the findings from few other studies which observed faster healing and decreased symptoms after PRP treatment in erosive OLP. ^27^^,^^28^ However, it was also noted that the included studies had small sample sizes, with a lack of blinding or placebo controls and short follow-up durations. Hence, future studies with more stringent protocols, large sample size and long term follow up are recommended.

### Strength and Limitations

This review has several strengths, including the comprehensive search strategy, rigorous inclusion criteria, and quantitative synthesis of outcomes where possible. Unlike earlier narrative or scoping reviews, our study conducts a quantitative meta-analysis, adding a higher level of evidence. ^29^^,^^30^

However, there are important limitations that must be acknowledged. Included studies showed small sample sizes, limited follow-up intervals, and variable PRP methods of production, which added clinical heterogeneity and affected the generalizability of the results. Direct comparisons were also difficult due to inconsistent result reporting and different methods of scoring used to evaluate clinical improvement.

## CONCLUSION

The results of our systematic review suggest that platelet-rich plasma is a potentially efficacious and safe treatment modality for oral lichen planus, specifically regarding alleviation of pain as well as mucosal healing improvement. However, due to methodological variation as well as the lack of high-quality evidence, further rigorous clinical trials must be carried out to confirm these inferences and establish standardized guidelines for treatment.

## References

[1] Talungchit S, Buajeeb W, Lerdtripop C, Surarit R, Chairatvit K, Roytrakul S, Kobayashi H, Izumi Y, Khovidhunkit SP (2018). Putative salivary protein biomarkers for the diagnosis of oral lichen planus: a case-control study. BMC Oral Health.

[2] Motahari P, Pournaghi Azar F, Rasi A (2020). Role of Vitamin D and Vitamin D Receptor in Oral Lichen Planus A Systematic Review. Ethiop J Health Sci.

[3] Veneri F, Bardellini E, Amadori F, Conti G, Majorana A (2020). Efficacy of ozonized water for the treatment of erosive oral lichen planus: a randomized controlled study. Med Oral Patol Oral Cir Bucal.

[4] Iocca O, Sollecito TP, Alawi F (2020). Potentially malignant disorders of the oral cavity and oral dysplasia A systematic review and meta-analysis of malignant transformation rate by subtype. Head Neck.

[5] Gururaj N, Hasinidevi P, Janani V, Divynadaniel T (2021). Diagnosis and management of oral lichen planus. Review. J Oral Maxillofac Pathol.

[6] Sridharan K, Sivaramakrishnan G (2021). Interventions for oral lichen planus A systematic review and network meta-analysis of randomized clinical trials. Aust Dent J.

[7] Pixley JN, Cook MK, Singh R, Larrondo J, McMichael AJ (2023). A comprehensive review of platelet-rich plasma for the treatment of dermatologic disorders. J Dermatolog Treat.

[8] Sriram S, Hasan S, Alqarni A, Alam T, Kaleem SM, Aziz S, Durrani HK, Ajmal M, Dawasaz AA, Saeed S (2023). Efficacy of Platelet-Rich Plasma Therapy in Oral Lichen Planus A Systematic Review. Medicina (Kaunas).

[9] Krishnamoorthy B, Khan M (2013). Management of oral submucous fibrosis by two different drug regimens A comparative study. Dental Research Journal.

[10] Patel TL, Singh S (2015). Comparative evaluation of treatment of Oral submucous fibrosis with intralesional injections of Dexamethasone and hyaluronidase with Triamcinolone Acetonide and hyaluronidase. Group.

[11] EL-Komy MH, Hassan AS, Raheem HM, Doss SS, EL-Kaliouby M, Saleh NA, Saleh MA (2015). Platelet-rich plasma for resistant oral erosions of pemphigus vulgaris A pilot study. Wound Repair and Regeneration.

[12] Hersle K, Mobacken H, Sloberg K, Thilander H (1982). Severe oral lichen planus treatment with an aromatic retinoid (etretinate). Br J Dermatol.

[13] Kakar PK, Puri RK, Venkatachalam VP (1985). Oral submucous fibrosis-treatment with hyalase. J Laryngol Otol.

[14] Maddheshiya N, Srivastava A, Rastogi V, Shekhar A, Sah N, Kumar A (2023). Platelet-rich plasma protein as a therapeutic regimen for oral lichen planus An evidence-based systematic review. Natl J Maxillofac Surg.

[15] Hydri AS, Udaipurwala IH, Sheikh NA, Sadiq SM, Aslam S (2020). Comparison of Triamcinolone Versus Platelet Rich Plasma Injection for Improving Trismus in Oral Submucous Fibrosis. J Bahria Univ Med Dent Coll.

[16] Sethi Ahuja U, Puri N, More CB, Gupta R, Gupta D (2020). Comparative evaluation of effectiveness of autologous platelet rich plasma and intralesional corticosteroids in the management of erosive oral Lichen planus- a clinical study. J Oral Biol Craniofac Res.

[17] Shah R, Triveni MG, Thomas R, Mehta DS (2017). An update on the protocols and biologic actions of platelet rich fibrin in dentistry. Eur J Prosthodont Restor Dent.

[18] Blanco J, Caramês J, Quirynen M (2025). A narrative review on the use of autologous platelet concentrates during alveolar bone augmentation Horizontal (simultaneous/staged) & vertical (simultaneous/staged). Periodontol 2000.

[19] Wang X, Yang Y, Zhang Y, Miron RJ (2019). Fluid platelet-rich fibrin stimulates greater dermal skin fibroblast cell migration, proliferation, and collagen synthesis when compared to platelet-rich plasma. J Cosmet Dermatol.

[20] Azizi B, Katebi K, Azizi H, Sarmadi MH (2024). Comparison of autologous platelet concentrates and topical steroids on oral lichen planus a systematic review and meta-analysis. BMC Oral Health.

[21] Bennardo F, Bennardo L, Del Duca E, Patruno C, Fortunato L, Giudice A (2020). Autologous platelet-rich fibrin injections in the management of facial cutaneous sinus tracts secondary to medication related osteonecrosis of the jaw. Dermatol Ther.

[22] Gasparro R, Adamo D, Masucci M, Sammartino G, Mignogna MD (2019). Use of injectable platelet-rich fibrin in the treatment of plasma cell mucositis of the oral cavity refractory to corticosteroid therapy a case report. Dermatol Ther.

[23] Mostafa D, Moussa E, Alnouaem M (2017). Evaluation of photodynamic therapy in treatment of oral erosive lichen planus in comparison with topically applied corticosteroids. Photodiagnosis Photodyn Ther.

[24] Xia J, Li C, Hong Y, Yang L, Huang Y, Cheng B (2006). Short- term clinical evaluation of intralesional triamcinolone acetonide injection for ulcerative oral lichen planus. J Oral Pathol Med.

[25] Andreasen JO (1968). Oral lichen planus 1. A clinical evaluation of 115 cases. Oral Surg Oral Med Oral Pathol.

[26] Roopashree MR, Gondhalekar RV, Shashikanth MC, George J, Thippeswamy SH, Shukla A (2010). Pathogenesis of oral lichen planus a review. J Oral Pathol Med.

[27] Córdova P, Rubio A, Echeverría P (2014). Oral lichen planus a look from diagnosis to treatment. J Oral Res.

[28] Shaik S, Jyothi PN, Kumar BV, Suman SV, Praveen KS, Sravanthi M (2020). Platelet rich plasma a new prospective in treatment of recalcitrant erosive lichen planus- a case report. Int J Res Rep Dent.

[29] Lee YC, Shin SY, Kim SW, Eun YG (2013). Intralesional injection versus mouth rinse of triamcinolone Acetonide in Oral lichen planus a randomized controlled study. Otolaryngol Head Neck Surg.

[30] Escudier M, Ahmed N, Shirlaw P (2007). A scoring system for mucosal disease severity with special reference to oral lichen planus. Br J Dermatol.

